# Neurosteroid 3α-Androstanediol Efficiently Counteracts Paclitaxel-Induced Peripheral Neuropathy and Painful Symptoms

**DOI:** 10.1371/journal.pone.0080915

**Published:** 2013-11-15

**Authors:** Laurence Meyer, Christine Patte-Mensah, Omar Taleb, Ayikoe Guy Mensah-Nyagan

**Affiliations:** Biopathologie de la Myéline, Neuroprotection et Stratégies Thérapeutiques, INSERM U1119, Fédération de Médecine Translationnelle de Strasbourg (FMTS), Université de Strasbourg, Faculté de Médecine, Strasbourg, France; University of Cincinnatti, United States of America

## Abstract

Painful peripheral neuropathy belongs to major side-effects limiting cancer chemotherapy. Paclitaxel, widely used to treat several cancers, induces neurological symptoms including burning pain, allodynia, hyperalgesia and numbness. Therefore, identification of drugs that may effectively counteract paclitaxel-induced neuropathic symptoms is crucial. Here, we combined histopathological, neurochemical, behavioral and electrophysiological methods to investigate the natural neurosteroid 3α-androstanediol (3α-DIOL) ability to counteract paclitaxel-evoked peripheral nerve tissue damages and neurological symptoms. Prophylactic or corrective 3α-DIOL treatment (4 mg/kg/2days) prevented or suppressed PAC-evoked heat-thermal hyperalgesia, cold-allodynia and mechanical allodynia/hyperalgesia, by reversing to normal, decreased thermal and mechanical pain thresholds of PAC-treated rats. Electrophysiological studies demonstrated that 3α-DIOL restored control values of nerve conduction velocity and action potential peak amplitude significantly altered by PAC-treatment. 3α-DIOL also repaired PAC-induced nerve damages by restoring normal neurofilament-200 level in peripheral axons and control amount of 2’,3’-cyclic-nucleotide-3’-phosphodiesterase in myelin sheaths. Decreased density of intraepidermal nerve fibers evoked by PAC-therapy was also counteracted by 3α-DIOL treatment. More importantly, 3α-DIOL beneficial effects were not sedation-dependent but resulted from its neuroprotective ability, nerve tissue repairing capacity and long-term analgesic action. Altogether, our results showing that 3α-DIOL efficiently counteracted PAC-evoked painful symptoms, also offer interesting possibilities to develop neurosteroid-based strategies against chemotherapy-induced peripheral neuropathy. This article shows that the prophylactic or corrective treatment with 3α-androstanediol prevents or suppresses PAC-evoked painful symptoms and peripheral nerve dysfunctions in rats. The data suggest that 3α-androstanediol-based therapy may constitute an efficient strategy to explore in humans for the eradication of chemotherapy-induced peripheral neuropathy.

## Introduction

Chemotherapy with antineoplastic drugs remains so far the widely used and unavoidable treatment of several cancers. However, the success of many cancer treatments is hampered by antineoplastic-induced painful peripheral neuropathy and neuropathological symptoms that constitute a major health concern. Neuropathic pain, histopathological, neurochemical and functional alterations of peripheral nerves evoked by anticancer drugs represent a leading cause of discontinuation of successful treatments [1,2]. These neuropathological symptoms restrict therapeutic doses of antineoplastics to suboptimal concentrations unable to kill tumor cells and significantly affect the quality of life of millions of patients [3-5]. Painful neuropathological symptoms are generated by various anticancer drugs including paclitaxel (also called taxol®) that is widely used to treat breast cancer and several other malignancies such as ovarian, lung, head and bladder cancers and AIDS-related Kaposi’s sarcoma [6-11]. Paclitaxel is known to induce sensory peripheral neuropathy with burning pain symptoms, allodynia, hyperalgesia tingling and numbness [1,3,12-18]. Several attempts have been made to treat or prevent antineoplastic-induced painful neuropathy with various neuroprotective agents but the results are contradictory and most of the compounds used were ineffective or induced themselves adverse effects such as nausea, reflex dysfunctions, treatment-emergent nervousness, insomnia, tremor, anorexia or stomach burning [19-23]. Therefore, it is crucial to identify novel drugs that may efficiently prevent or suppress antineoplastic-induced painful and neuropathological symptoms without evoking themselves undesirable action or severe side-effects. In a recent study, we observed that progesterone-derived 3α-5α-reduced metabolites exerted potent neuroprotective effects against oxaliplatin- or vincristine-induced peripheral neuropathy in rats, suggesting that 3α-5α-neurosteroid-based strategies may offer serious opportunities to develop effective therapies against anticancer drug-evoked painful neuropathic symptoms [24,25]. Among the family of 3α-5α-neurosteroids are naturally-produced molecules such as allopregnanolone and 3α-androstanediol (3α-DIOL) which activate allosterically the GABA_A_ receptor and potentiate the central inhibition [26-34]. While allopregnanolone has extensively been investigated in several experimental models and in humans as a neuroprotective, neurogenic, anaesthetic, anxiolytic or analgesic compound, the neurosteroid 3α-DIOL has received little attention [26,35-42]. The present study was therefore designed to investigate whether 3α-DIOL may also be a neuroprotective neurosteroid offering effective possibilities for the treatment of chemotherapy-induced painful neuropathy and neuropathological symptoms. Thus, we assessed the potential of 3α-DIOL to prevent or suppress paclitaxel-evoked neurochemical and functional abnormalities in rat peripheral nerves. In particular, we used a multidisciplinary approach to determine the ability of 3α-DIOL to prevent or counteract several neuropathological parameters evoked by paclitaxel therapy such as behavioral painful symptoms (mechanical and thermal allodynia or hyperalgesia), electrophysiological deficiency (decreased nerve conduction velocity) and histopathological alterations including the repression of neurofilament 200 kDa (NF200) in peripheral nerve axons, the decrease of intraepidermal nerve fiber (IENF) density in intraplantar skin and the reduction of peripheral expression of myelin enzyme 2’,3’-cyclic-nucleotide-3’-phosphodiesterase (CNPase) that is pivotal for axonal survival [43,44].

## Methods

### Animals

Adult male Sprague-Dawley rats weighing 250-300 g were used. The experiments were performed with male animals in order to avoid eventual fluctuations of results that may happen in females through the different phases of the oestral cycle [45-47]. The animals were obtained from a commercial source (Janvier, Le Genest St Isle, France) and housed under standard laboratory conditions in a 14/10 h light/dark cycle with food and water *ad libitum*. Animals were allowed a 1 week acclimatization period before being used in experiments.

### Ethic statement

Animal care and manipulations were performed according to The European Community Council Directives (86/609/EC) and under the supervision of authorized investigators. All experiments were performed minimizing the number of animals used and their suffering in accordance with the Alsace Department of Veterinary Public Health Guide for the Care and Use of Laboratory Animals. The ethics committee of the Alsace Department of Veterinary Public Health Guide for the Care and Use of Laboratory Animals specifically approved the protocol used in the present study (Agreement number 67-186). The experiments also followed the International Association for the Study of Pain ethical guidelines [48].

### Drugs and treatments

Paclitaxel (PAC; Sequoia Research Products Ltd, UK) was dissolved in 10% Cremophor EL^®^ in saline (Sigma-Aldrich, St Louis, MO, USA), used as vehicle (VEH_crem_) and stored at 4°C. PAC was intraperitoneally (i.p.) injected every-2 days during 1 week, at a concentration of 1 mg/kg/injection depending on the daily body weight [16]. The chemotherapy was therefore performed at D1, D3, D5 and D7. Thus, the animals received a total of 4 mg/kg of PAC. Control rats were injected with the vehicle ‘10% Cremophor EL^®^ in saline’ solution or VEH_crem_ (1 ml/kg) according to a similar schedule. Before the onset of PAC (versus vehicle) treatment, behavioral tests were performed in all rats in order to determine the pre-injection values of the mechanical (Day 0 or D0), cold (D0) and heat thermal (Day 1 or D1) nociceptive thresholds. After PAC or vehicle treatment onset, behavioral analyses were realized once a week to assess alternately the mechanical or thermal sensitivity.

3α-androstanediol or 3α-DIOL (Steraloids, Newport, RI, USA) was diluted in hydroxypropylcellulose or HPC 0.3% (Sigma-Aldrich, St Louis, MO) in water used as vehicle (VEH_hpc_). Consequently, it should be noted that 4 different groups of control animals were used for the whole study: (i) naive non-treated rats, (ii) (VEH_crem_)-treated rats, (iii) (VEH_hpc_)-treated rats and (iv) (VEH_crem_ + VEH_hpc_)-treated rats. 3α-DIOL was i.p. administered immediately after the behavioral test session. Two types of treatments were performed using 3α-DIOL. Corrective 3α-DIOL treatment, which aimed at suppressing painful neuropathic symptoms induced by the 1 week PAC treatment (administered at D1, D3, D5, and D7), consisted in starting 3α-DIOL i.p. administration 8 days after the end of PAC treatment (D15). Therapy with 3α-DIOL lasted 2 weeks (D15, D17, D19, D21, D23, D25 and D27). Prophylactic 3α-DIOL treatment, which aimed at preventing the development of PAC-induced neuropathic pain, consisted in 3α-DIOL administration before the onset and during the treatment with PAC. Thus, for the prophylactic strategy (3α-DIOL administration before PAC injections), 3α-DIOL was administered at D1, D3, D5 and D7 before PAC treatment started at D8. Then, PAC was injected at D8, D10, D12 and D14 while 3α-DIOL treatment was continued at D9, D11, D13, D15, D17, D19, D21, D23 and D25. Behavioral experiments, electrophysiological studies, immunohistochemical investigations, microscopic and cellular analyses were performed to determine 3α-DIOL effects on PAC-induced painful symptoms as well as peripheral nerve alterations. Experimenters were completely blind to the experimental conditions of animals. Corrective and prophylactic effects of 3α-DIOL against PAC-induced painful neuropathy were investigated along the treatment period but also one week after the end of 3α-DIOL administration.

### Nociceptive behavioral tests

*The mechanical nociceptive sensitivity* threshold was evaluated in individual rats placed on Plexiglas® boxes (30 x 30 x 25 cm) upon an elevated metal grid allowing access to the plantar surface of the hind paws. The presence of mechanical allodynia and hyperalgesia were assessed using a series of calibrated von Frey hairs (1, 2, 4, 6, 8, 10, 15, 26g; Stoelting, Wood Dale, IL), which were applied to the plantar surface of the hind paw with increasing force until the individual filament used just started to bend. The filament was applied for a period of 1-2 s and the procedure was repeated five times at 4-5 s intervals. The threshold for paw withdrawal was calculated by taking the average of ten (five per paw) repeated stimuli (in g) which induced a reflex paw withdrawal. Only robust and immediate withdrawal responses followed by a licking of the paw were considered as positive. Naive untreated rats never withdraw from stimulations less than 6 g but respond 15-20% of the time for 15 g stimulus and 35-40% for 26 g stimulus. Observation of responses for stimulations < 6 g after drug administrations is indicative of mechanical allodynia. Increased level of responding for 15 and 26 g after treatment is indicative of mechano-hyperalgesia. Decreased level of responding for 26 g after treatment is indicative of antinociceptive effect.

*Heat thermal nociceptive sensitivity* was assessed by using a Plantar test apparatus (Ugo Basile, Comerio, Italy) which measures the paw withdrawal latency in response to radiant heat [49]. The rats were first allowed to habituate to the apparatus for 10 min before testing. Each rat was placed individually in clear Plexiglas® boxes (23 x 18 x 14 cm) positioned on a clear plastic surface. The heat source was then positioned under the plantar surface of the hind paw and activated with an infra-red light beam. The heat source is connected to a timer which automatically switched off the heat when the paw was withdrawn. A cut-off time of 20 s was used to prevent tissue damage in absence of response. Three latencies were obtained alternately from each hind paw 5 min apart. No consistent left and right differences were observed. The mean paw withdrawal latency (in seconds) of hind paws was determined from an average of six separate measures (three per paw) at a given time point. Testing boxes were thoroughly cleaned between each test session.

*Cold thermal nociceptive sensitivity* was assessed by using the acetone test. Rats were placed on Plexiglas® boxes (30 x 30 x 25 cm) upon an elevated metal grid allowing access to the plantar surface of the hind paws. Fifty microliters of acetone (Sigma-Aldrich, St Louis, MO) was sprayed onto the plantar skin of each hind paw 3 times with a Hamilton syringe. Rats were observed 20 s from the start of the acetone spray. The latency is measured for the withdrawal followed by a licking of the hind paw. Normal rats ignore the stimulus or occasionally respond with a very small withdrawal taking place lately without licking the hind paw. Neuropathic animals respond with a rapid and intense withdrawal followed by a licking of the hind paw.

Three latencies were obtained alternately from each hind paw 5 min apart. No consistent left and right differences were observed. The mean paw withdrawal latency (in seconds) of hind paws was determined from an average of six separate measures (three per paw) at a given time point. Testing boxes were thoroughly cleaned between each test session.

### Openfield test

The openfield (100x100x35 cm) has a 64-square grid floor (8x8 squares, 12.5 cm/side) with an overhead light for illumination. The number of squares entered and the number of rears on the wall were used as indexes of horizontal and vertical exploratory behavior, respectively. These numbers were recorded during a five-minute test period.

### Electrophysiological studies

Sciatic nerve action potential (NAP) recording was performed as previously described [24,25]. Briefly, sciatic nerves were rapidly dissected from decapitated rats under urethane anesthesia (25%, 0.5 ml/100g) and immerged in saline medium (NaCl 133, KCl 2, CaCl_2_ 1, MgCl_2_ 2, HEPES 10 and glucose 10 pH 7.4) 5 to 10 min before recording. The sciatic nerve was put on chlorinated silver electrodes mounted in a classical homemade electrodes holder chamber. Square-shaped stimulating pulses of 0.1ms duration generated by Clampex routine of the Pclamp software package (Axon Instruments, CA, USA) were applied to the distal end of the nerve through Digidata 1322A interface (Axon Instruments, CA, USA). Bipolar sciatic nerve action potentials (NAP) were recorded at the proximal end of the nerve (∆x=19 mm distance from the stimulus point) using an ISO-DAM8A differential amplifier (Word Precision Instruments, UK) with a bandwidth of 10 - 10,000 Hz. The signal was digitized (500 kHz) with the Digidata 1322A. Artifact of stimulation was obtained in isolation using a double pulse protocol allowing the second stimulation to occur in refractory period. This stimulus artifact was subtracted from recorded NAPs before analysis with Pclamp software.

Nerve conduction velocity (CV) was calculated using the latency between the beginning of stimulus artifact and the NAP onset (∆t_Latency_) or the NAP peak (∆t_Peak_) for the fastest and relatively slower fibers group respectively according to the equations:

CV_Latency_ = ∆x / ∆t_Latency_

CV_Peak_ = ∆x / ∆t_Peak_

Data obtained from the different conditions were compared using one way ANOVA statistical test followed by Bonferroni's Multiple Comparison Test. Statistical significance was fixed as usually at *p*<0.05.

### Immunohistochemical studies

At the end of the last behavioral test session, sciatic nerves and hind paw intra-plantar skins were removed to allow immunohistochemical studies. Thus, animals were deeply anesthetized with 25% urethane (0.5 ml/100g, i.p.) and perfused transcardially with 100 ml of 0.1 M phosphate buffered (PB; pH 7.4). The perfusion was carried out with 450 ml of fixative solution (4% formaldehyde and 0.2% picric acid in PB). Sciatic nerves and plantar skins were rapidly dissected and postfixed in the same fixative solution for 24h. Afterwards, the tissues were immersed in PB containing 15% sucrose for 24h and then transferred into 30% sucrose PB for 24h. Sciatic nerves and intraplantar skins were then placed in embedding medium (OCT, Tissue-Tek, Reichert-Jung, Nussloch, Germany) and immediately frozen at -80°C. Sagittal sections (10-µm thick) were cut in a cryostat HM 560 (Microm, Francheville, France) and mounted on glass slides coated with gelatin and chromium potassium sulfate. Tissue sections were pre-incubated for 1 hour with 5% nonimmune donkey or goat serum in PB containing 0.3% Triton X-100 (PBT). Afterwards, the sciatic nerve sections were incubated overnight at 4°C with a mouse monoclonal antibody against NF200 (Clone N52; Sigma-Aldrich, St Louis, MO) diluted at 1:250 in PBT or a mouse monoclonal antibody against 2’,3’-cyclic nucleotide 3’-phosphodiesterase (CNPase; Sigma-Aldrich, St Louis, MO) diluted at 1:100 in PBT. Hind paw intra-plantar skin sections were incubated overnight at 4°C with the rabbit polyclonal antibody against the protein gene product PGP9.5 (Cedarlane Laboratories, Ontario, Canada) diluted at 1:500 in PBT. The procedure was carried on by rinsing all of the tissue sections 3 times in PB (15 min/rinse) and transferring them for 2 hours into Alexa-488-conjugated donkey anti-mouse or FITC-conjugated goat anti-rabbit (Chemicon, Temecula, CA) diluted at 1:300 in PBT. Finally, the sections were rinsed for 1 hour in PB and mounted in Vectashield (Vector Laboratories, Burlingame, CA). Although specificity of the antibodies has previously been demonstrated [50-54], internal control experiments were performed in the present work as follows: (i) substitution of CNPase, NF200 or PGP9.5 antiserum with PBT, (ii) replacement of CNPase, NF200 or PGP9.5 antibody by non-immune mouse or rabbit serum and (iii) preincubation of CNPase, NF200 or PGP9.5 antibody with purified CNPase, NF200 or PGP9.5, respectively. The preparations were examined under a multichannel confocal laser-scanning microscope (Leica Confocal Systems, Paris, France) assisted by a pentium IV PC (Leica Microsystems). The number of CNPase or NF200 immunoreactive fibers was determined in a counting square of 200 x 200 µm^2^ on sagittal sections of sciatic nerves isolated from each experimental group. Quantification of NF200 immunoreactivity was performed on sciatic nerve sagittal sections by using ImageJ software. Results were expressed as % of control (+ SEM). The number of PGP9.5 positive-intraepidermal nerve fibers (IENF) was quantified as previously described [50,52]: all ascending nerve fibers that cross into the epidermis were counted, no minimum length was required and secondary branching into the epidermis was excluded from quantification. IENF counts are expressed as the number per mm of epidermal border. Experimenters were completely blind to the experimental conditions of animals.

### Statistical analyses

One or two-way repeated measures ANOVAs followed by Newman-Keuls *post hoc* comparisons were used. The data which did not exhibit a Gaussian distribution were analyzed by the non-parametric Mann-Whitney *U* test. The statistical significance of differences between sciatic nerve conduction velocities or NAP amplitude peaks was assessed by one way ANOVA statistical test followed by Bonferroni's Multiple Comparison Test. Data were analyzed with Statistica Software 5.1 (Statsoft, Maisons-Alfort, France). A *p*-value of less than 0.05 was considered significant.

## Results

### Effects of PAC on the mechanical nociceptive threshold

**Figure 1:**
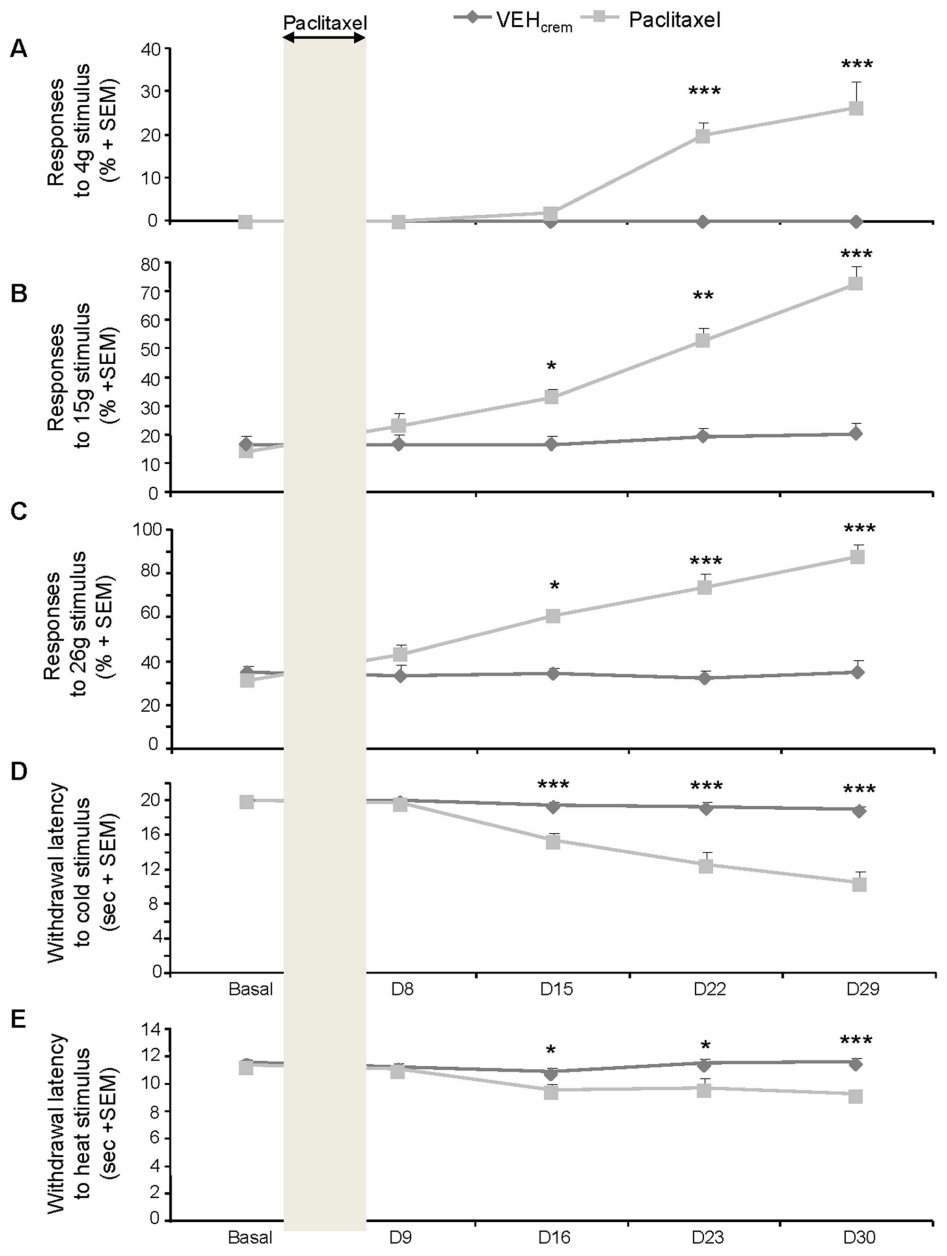
Effect of PAC treatment on the rat mechanical (A-C) and thermal (D, E) nociceptive threshold. (**A**-**C**) Time-course of mechanical allodynia (**A**) and hyperalgesia (**B**,**C**) induced by PAC treatment. Graphs show the mean + SEM of the percentages of paw withdrawal responses to mechanical stimulation by von Frey filament 4 g (**A**), 15 g (**B**) or 26 g (**C**) (n=8 per group). (**D**,**E**) Effects of PAC treatment on rat cold (**D**) or heat (**E**) thermal nociceptive threshold assessed by acetone (**D**) or Plantar (**E**) tests. Each point represents the mean + SEM of 6 observations in each of 8 rats. Non-parametric Mann-Whitney *U* test was used for the analysis of the von Frey test results and one-way repeated measures ANOVAs followed by Newman-Keuls *post* *hoc* comparisons were used for acetone and Plantar tests. Statistical differences between control and paclitaxel group on each testing day are shown. * *p*<0.05, *** *p*<0.005.

Before the onset of PAC or vehicle (Cremophor 10%) treatment, the percentages of paw withdrawal for mechanical stimulations were 0% for von Frey filaments < 6 g, 10-15% for 15 g and 35-40% for 26 g. In animals receiving the vehicle, the percentages of withdrawal responses remained unchanged all treatment days (Figure 1A-C). In contrast, from D15, paw withdrawal responses in PAC-treated rats were observed (3-26%; *p*<0.005) for von Frey filaments < 6 g (mechanical allodynia) (Figure 1A) and increased levels of responding for 15g (30-71.5%; *p*<0.005) and for 26 g (60-87.5%; *p*<0.005) were detected (mechano-hyperalgesia) (Figure 1B-C). PAC-induced mechanical allodynia and hyperalgesia, which started one week after the withdrawal of PAC treatment, persisted, at least 3 weeks (Figure 1A-C).

### Effects of PAC on cold thermal nociceptive sensitivity

Prior to PAC or vehicle (10% Cremophor in saline) treatment, no paw withdrawal response was induced by acetone spray during the whole observation period (20 s), indicating that acetone-evoked cold stimulation is not noxious in naive rats. In animals receiving the vehicle, the withdrawal latency remained unchanged (no paw withdrawal response during 20 s) all treatment days (Figure 1D). In contrast, the withdrawal latency significantly decreased from 20 s to 15 s at D15 and to 10 s at D29 (last time point recorded) in PAC-treated animals (*p*<0.001), showing that PAC treatment caused cold thermal allodynia in rats (Figure 1D).

### Effects of PAC on heat thermal nociceptive sensitivity

The baseline withdrawal latency characterizing the heat thermal pain threshold was around 11.5 ± 0.2 s on each paw of naive rats before the treatment onset. No significant changes of the thermal thresholds were observed in animal receiving the vehicle (Figure 1E). In contrast, a significant decrease of the heat thermal nociceptive threshold was observed in PAC-treated rats (from 11.4 to 9.2 s) at D16 (*p*<0.05) and this threshold remained significantly lower until D30 (*p*<0.001) (Figure 1E).

### Effects of PAC on sciatic nerve action potential and conduction velocity

**Figure 2:**
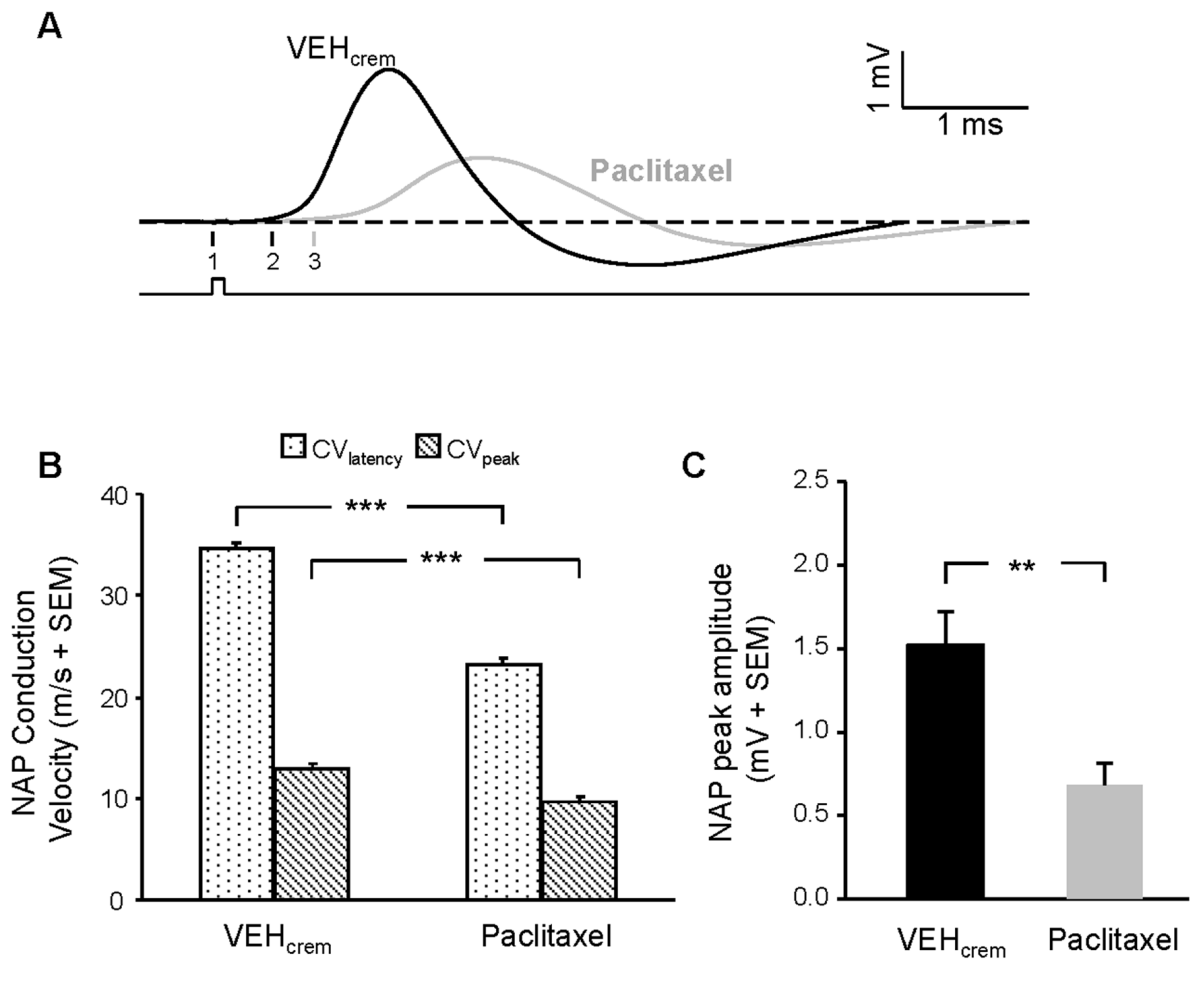
PAC effects on rat sciatic nerve action potential (NPA) peak amplitude and conduction velocity. (**A**) Mean traces of biphasic NAPs recorded from animals treated with vehicle (*black curve*) or PAC (*grey curve*). The nerve was stimulated with a supra-maximal pulse potential (1 V) as indicated by the protocol trace (*lower trace*). The arrows 1-3 indicate the beginning of the stimulation artifact (1) and the onset of NAP in control (2) and PAC (3) conditions giving a calculated NAP CV_latency_ in this case of 39.6 and 23.2 m/s for control and PAC, respectively. Note the reduction of NAP peak amplitude in PAC condition (1.2 mV versus 2.9 mV in controls). (**B**,**C**) NAP conduction velocity (**B**) and peak amplitude (**C**) histograms of statistical data obtained for vehicle- and PAC-treated rats (n=8 for each condition). ** *p*<0.01, *** *p*<0.005.

The sciatic NAP recorded in our conditions concern the myelinated fibers that may essentially be Aδ fibers owing to their conductance velocity range. As these fibers have different diameters, a large bandwidth of CVs is generated. The higher fiber CVs contribute to the NAP onset while the smallest CVs are involved in the NAP peak. Figure 2A illustrates mean traces of sciatic NAPs recorded from vehicle- and PAC-treated rats. PAC affected both NAP peak amplitude and conductance velocity. In the case illustrated in Figure 2A, the CV of the fastest fibers (NAP onset) was reduced by 41.4% under PAC treatment (39.6 and 23.2 m/s for vehicle and PAC, respectively) while the slowest fibers (peak of NAP) was decreased about 33.6% (absolute values at NAP peak CV were 13.3 and 8.8 m/s in vehicle and PAC treated rats, respectively). Mean reduction values obtained for CV_latency_ and CV_peak_ were 32.9 ± 0.7 and 24.7 ± 1.0%, respectively (Figure 2B). The NAP peak amplitude was greatly reduced and the values for the NAP’s first phase in the case illustrated were 2.9 and 1.2 mV for vehicle- and PAC-treated rats, respectively, giving a 58% inhibition of sciatic nerve function (Figure 2A). Mean values obtained in each condition were 1.5 ± 0.2 and 0.7 ± 0.1 mV respectively for vehicle- and PAC-treated rats, indicating that the mean decrease of NAP peak amplitude evoked by PAC was 54 ± 3% (Figure 2C).

### Effects of PAC on NF200 expression in peripheral nerve axons

**Figure 3:**
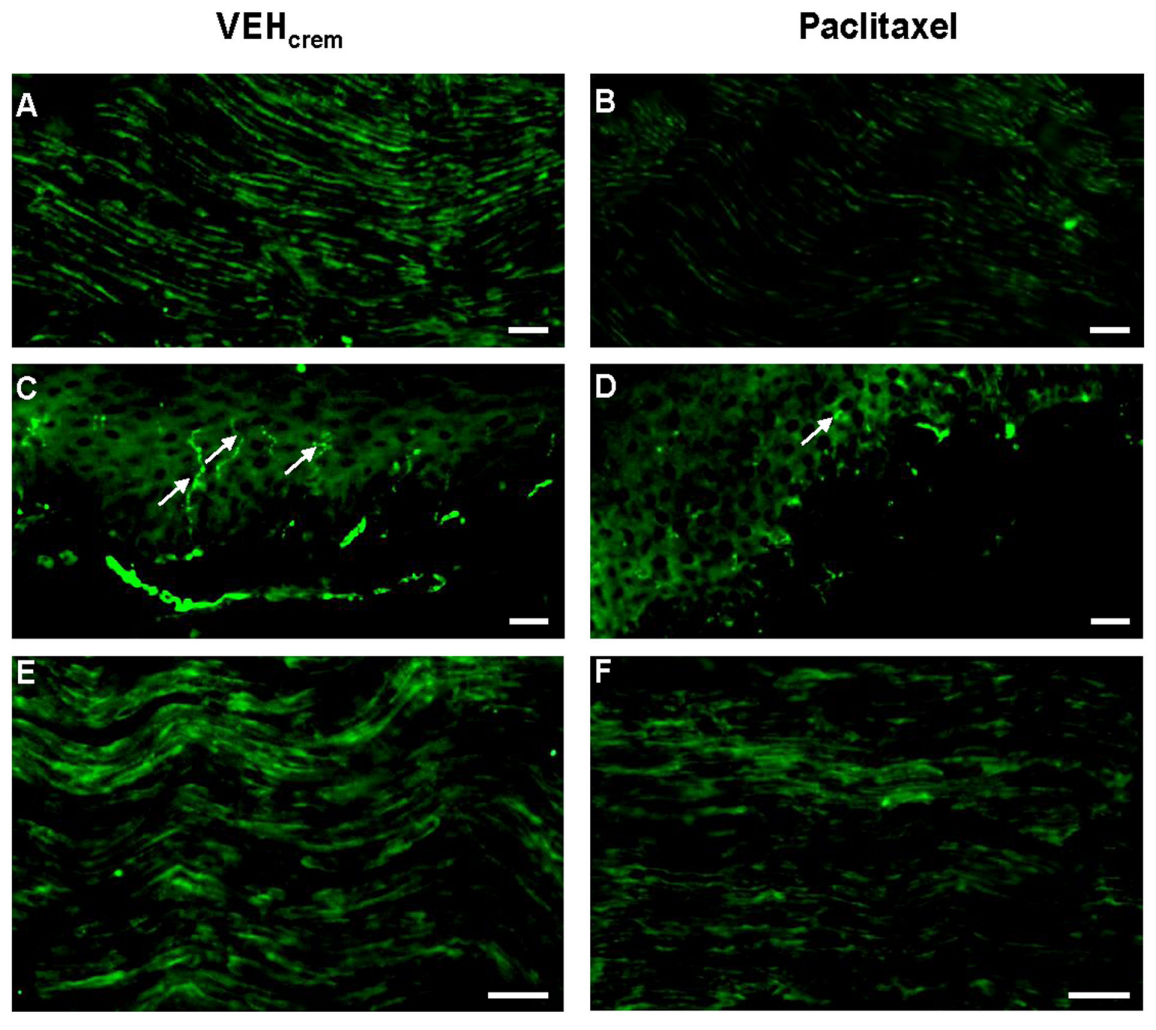
Photomicrographs of sagittal sections of sciatic nerves (A,B,E,F) or hind paw intraplantar skins (C,D) dissected from vehicle (A,C,E)- and PAC (B,D,F)-treated rats. Nerve sections were labeled with the monoclonal NF200 antibody (**A**,**B**) or with the monoclonal anti-CNPase (**E**,**F**) revealed with Alexa-488-conjugated donkey anti-mouse. (**C**,**D**) Intraplantar skin sections were labeled with the polyclonal anti-PGP9.5 revealed with FITC-conjugated goat anti-rabbit. White arrows indicated intraepidermal nerve fibers. Scale bar=50µm.

**Figure 4:**
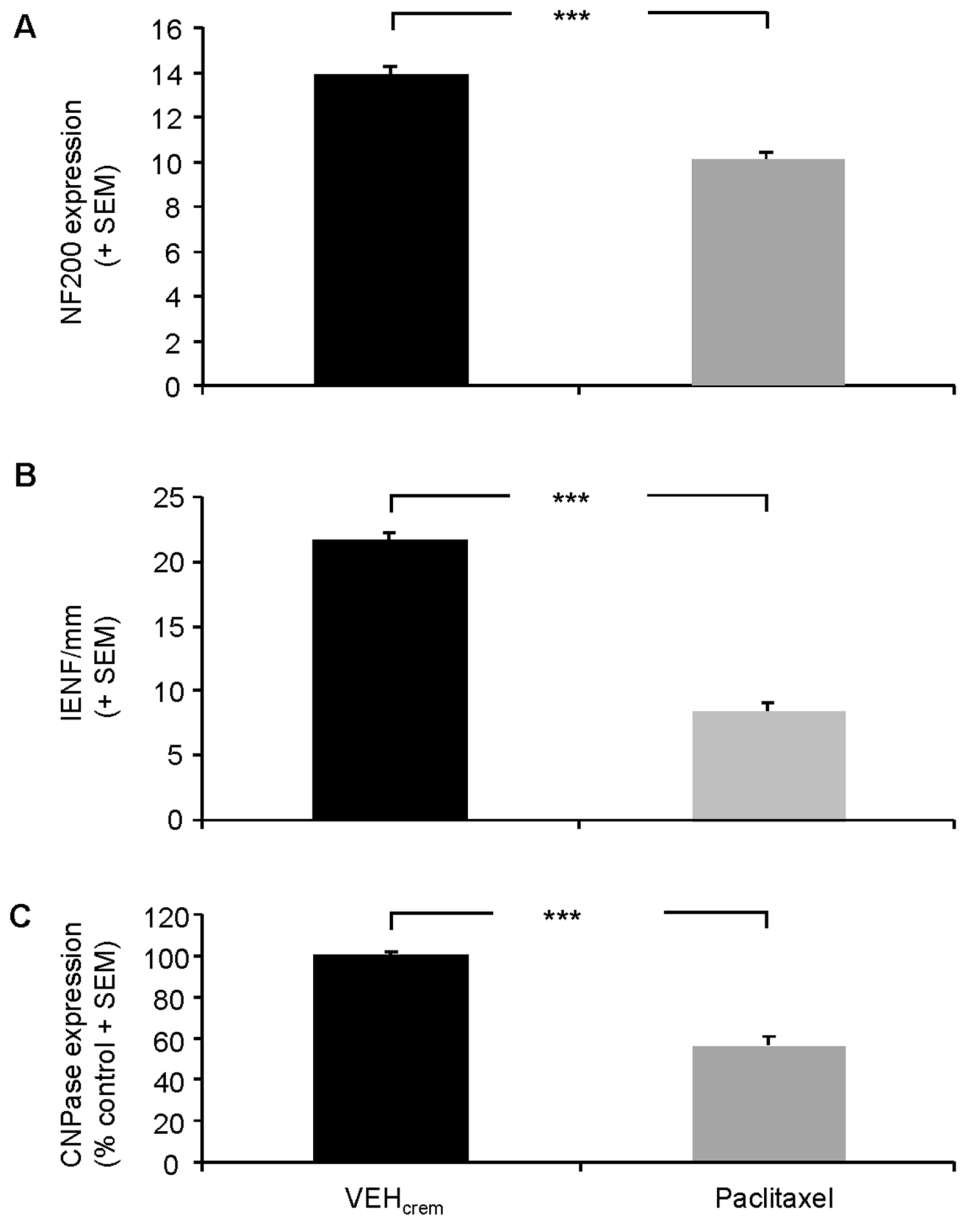
Comparison of IENF density, NF-200 or CNPase expression in vehicle- and PAC-treated rat nerves. (**A**) Comparative analysis of NF200-immunostaining density (actual counts for NF200-positive fibers) detected in sciatic nerve sections dissected from vehicle- and PAC-treated rats. (**B**) Comparative analysis of IENF density (counts for PGP9.5-positive terminals) measured in vehicle- and PAC-treated rat intraplantar skin sections. (**C**) Comparative analysis of the numbers of CNPase-positive Schwann cell bodies detected in sciatic nerve sections dissected from vehicle- and PAC-treated rats. Each value is expressed as percent (+ SEM) of CNPase-positive cells bodies detected in sciatic nerve sections of control (vehicle-treated) rats. n=8 per group. *** *p*<0.001.

To determine whether PAC treatment alters the structural organization of peripheral axons, we used immunohistochemical procedure to compare the expression of the key neuronal cytoskeleton protein NF200 [55] in PAC-treated and control rat sciatic nerve axons. Our results showed an intense NF200-like immunoreactivity in several processes in vehicle-treated or control rat sciatic nerves (Figure 3A). PAC-treatment drastically decreased NF200-immunofluorescence in sciatic nerve axons (Figure 3B). Quantitative analysis using the counting score method combined with ImageJ software calculation revealed a 33% decrease of NF200-immunostaining in PAC-treated sciatic nerve axons compared to controls (*p*<0.001) (Figure 4A).

### Effects of PAC on intraepidermal nerve fiber density in intraplantar skin

Intraepidermal nerve fibers (IENF) were firstly immunolabeled in rat hind paw intraplantar skin with a specific antibody against PGP9.5 (Figure 3C,D). Then, IENF density was quantified in vehicle- and PAC-treated rat hind paw intra-plantar skins. PAC treatment decreased by 61% PGP9.5-immunoreactivity or IENF density in intra-plantar epidermis (*p*<0.001) (Figure 4B).

### Effects of PAC on CNPase expression in sciatic nerves

As previous studies demonstrated that the non-compact myelin protein CNPase, pivotal for axonal survival, is dysregulated in experimental peripheral neuropathies [24,43,44], we have determined the effects of PAC treatment on CNPase expression in Schwann cells surrounding axonal processes of the rat sciatic nerve. Intense immunoreactivity for CNPase was visualized on sagittal sections of naive or vehicle-treated rat sciatic nerves (Figure 3E). PAC-treatment significantly reduced the intensity of CNPase-immunostaining in sciatic nerves (Figure 3F). Comparative analysis of the numbers of CNPase-positive Schwann cell bodies detected in counting squares (200 x 200 µm^2^) showed that PAC treatment caused a drastic decrease (-44%) of CNPase expression in sciatic nerves (*p*<0.001) (Figure 4C).

### Action of 3α-androstanediol against PAC-induced cold allodynia, heat hyperalgesia and mechanical allodynia or hyperalgesia

**Figure 5:**
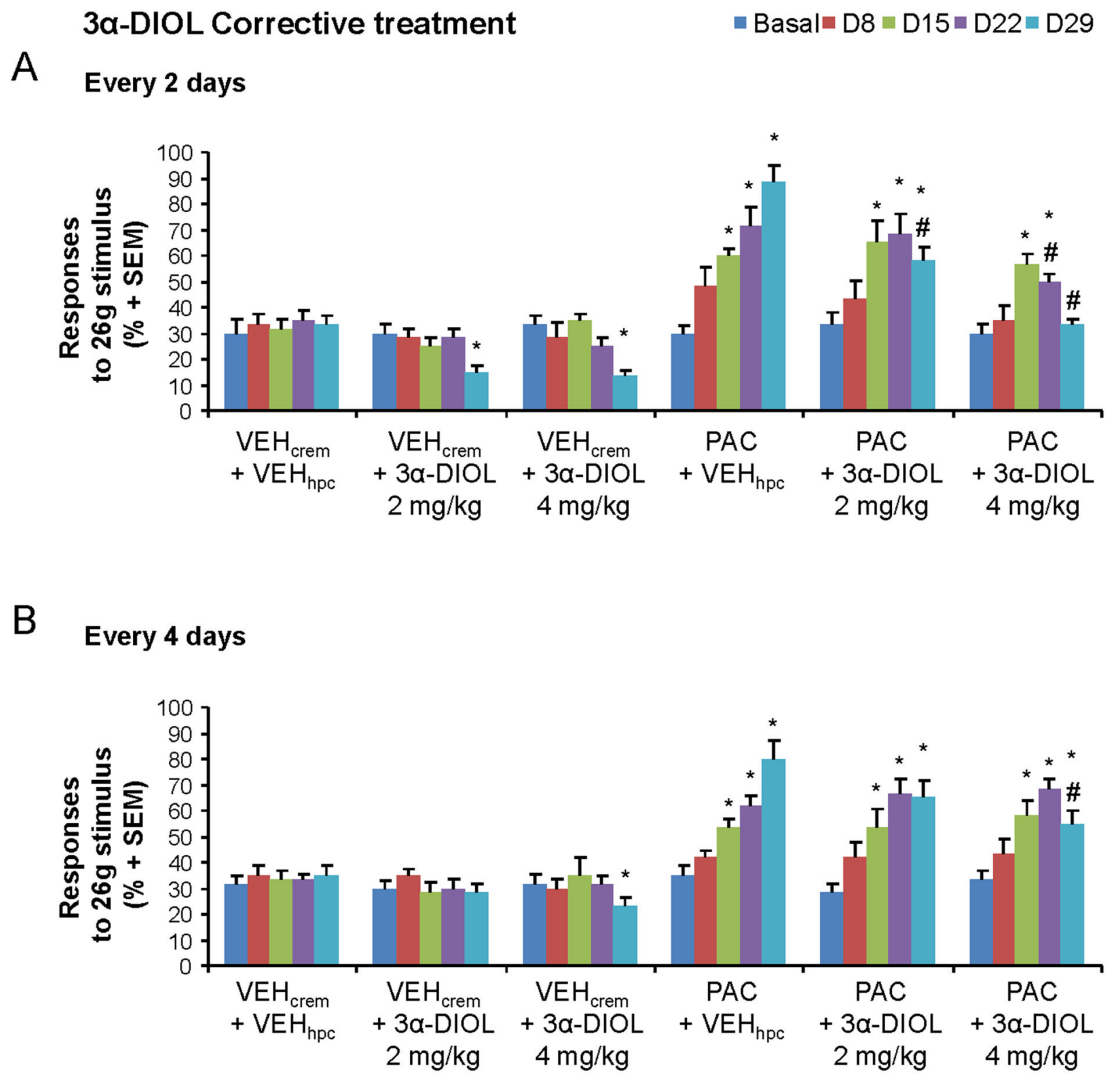
Dose- and injection frequency-dependent effects of corrective 3α-DIOL treatment on the mechanical nociceptive thresholds of control- and PAC-treated rats. Corrective treatment every 2- (**A**) or 4-days (**B**) consisted in starting 3α-DIOL (2 or 4 mg/kg) or VEH_hpc_ i.p. administrations 2 days after the end of PAC treatment. Threshold values represent responses to 26 g von Frey filament (% ± SEM). Non-parametric Mann-Whitney *U* test was used. Statistical differences between controls and each treatment group at each testing day are shown. n=6 per group; * *p*<0.05. * compared to VEH_crem_+VEH_hpc_; # compared to PAC+VEH_hpc_.

**Figure 6:**
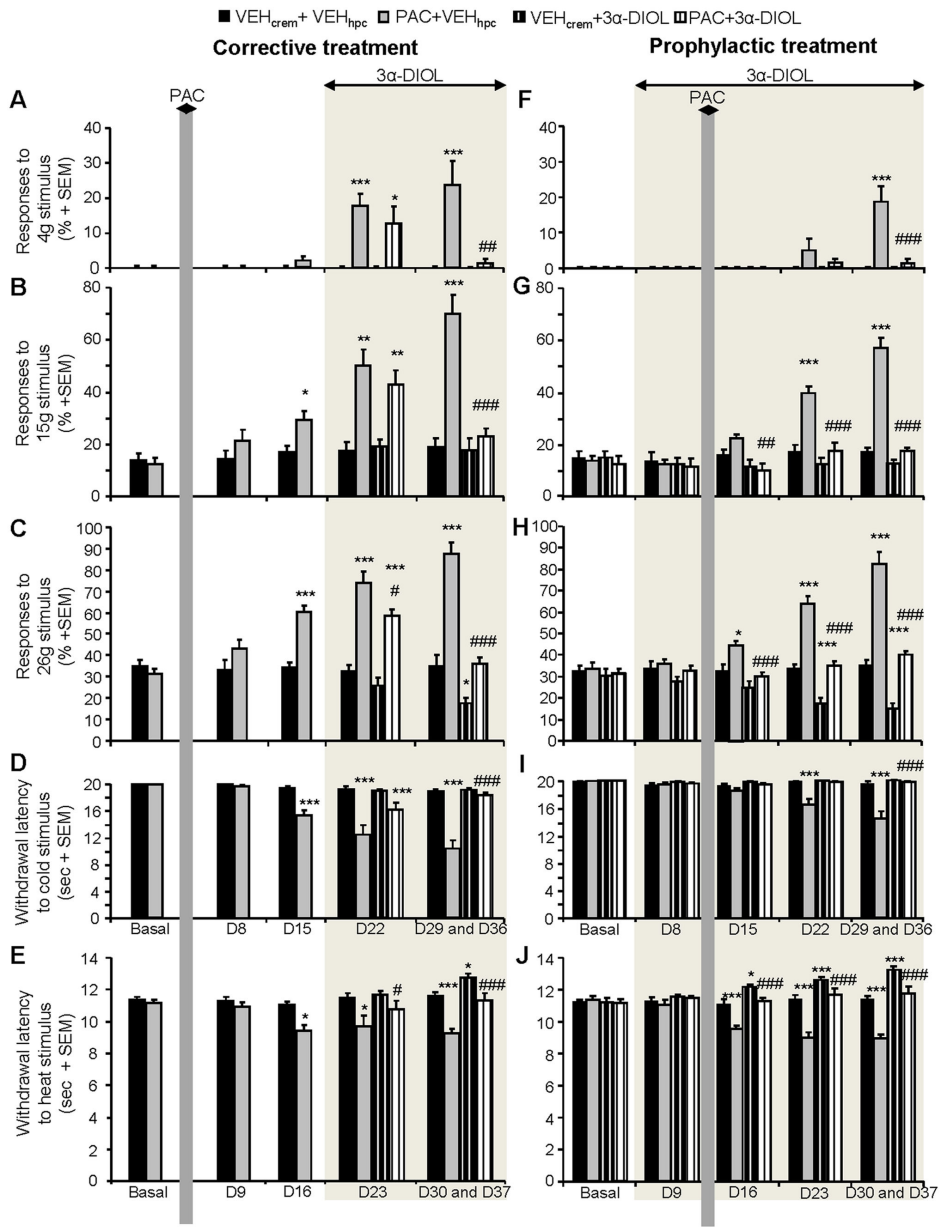
Effects of 3α-DIOL (4 mg/kg/2 days) corrective (A-E) or prophylactic (F-J) treatment on PAC-induced neuropathic pain symptoms. (**A**-**C**,**F**-**H**) Action of 3α-DIOL against PAC-induced mechanical allodynia (**A**,**F**) and hyperalgesia (**B**,**C**,**G**,**H**). Chartbars show the mean + SEM of the percentages of paw withdrawal responses to mechanical stimulation by von Frey filament 4 g (**A**,**F**), 15 g (**B**,**G**) or 26 g (**C**,**H**) (n=8 per group). (**D**,**I**) Effect of 3α-DIOL against PAC-evoked cold-allodynia. (**E**,**J**) 3α-DIOL effects on the heat thermal nociceptive thresholds of vehicle- and PAC-treated rats. Each bar represents the mean + SEM of 6 observations in each of 8 rats. Non-parametric Mann-Whitney *U* test was used for the analysis of the von Frey test results and two-way repeated measures ANOVAs followed by Newman-Keuls *post* *hoc* comparisons were used for acetone and Plantar Tests. Statistical differences between controls and each treatment group at each testing day are shown. * *p*<0.05, ** *p*<0.01, *** *p*<0.005. * vs (VEH_crem_ + VEH_hpc_), # vs (PAC + VEH_hpc_).

Although previous dose-response studies have identified 1 to 4 mg/kg as an effective dose range for 3α-androstanediol neuroactivity *in vivo* [29,32,56,57], we investigated in the present work the dose- and injection frequency-dependent effects of 3α-DIOL (by testing 2 or 4 mg/kg every 2 or 4 days) on the mechanical nociceptive thresholds of vehicle- and PAC-treated rats (Figure 5). In vehicle-treated animals, corrective 3α-DIOL treatments at 2 or 4 mg/kg every 2 days (Figure 5A) or at 4 mg/kg every 4 days (Figure 5B) significantly decreased the percent of responses to 26 g (*p*<0.05), indicating an antinociceptive effect of 3α-DIOL. In PAC-treated animals, the mechanical hyperalgesia (*p*<0.05) evoked by PAC was only partially reduced by 3α-DIOL corrective treatment at the dose of 2 mg/kg every 2 or 4 days or at 4 mg/kg every 4 days (*p*<0.05; Figure 5). In contrast, at the dose of 4 mg/kg every 2 days, 3α-DIOL corrective treatment was capable to abolish completely PAC-induced mechano-hyperalgesia and to restore a normal mechanical sensitivity threshold in PAC-treated rats (*p*<0.05; Figure 5A). Interestingly, we observed that the prophylactic treatment with 3α-DIOL at the same dose (4 mg/kg/2 days) has also successfully prevented the occurrence of PAC-evoked abnormal mechanical perception (Figure 6). Consequently, 3α-DIOL at 4 mg/kg injected every 2 days appeared as the best or optimal dose to be considered for the elaboration of an effective corrective or prophylactic strategy against PAC-induced neuropathic symptoms in rats. Indeed, 3α-DIOL (4 mg/kg/2 days), which increased the mechanical and thermal pain thresholds (*p*<0.05), exerted a potent antinociceptive action in control rats (Figure 6C,E,H,J). When neuropathic pain symptoms are already installed in PAC-treated rats, corrective 3α-DIOL treatment administered during two weeks (7 injections of 3α-DIOL 4 mg/kg) totally abolished the cold allodynia, heat hyperalgesia as well as the mechanical allodynia and hyperalgesia (*p*<0.005) by restoring normal cold- and heat-thermal and mechanical pain thresholds in PAC-treated animals (Figure 6A-E). Prophylactic administration of 3α-DIOL (4 mg/kg every 2 days), which started 8 days before PAC injections and was maintained during PAC treatment, completely prevented PAC-induced cold allodynia, heat hyperalgesia and mechanical allodynia or hyperalgesia (*p*<0.005) (Figure 6F-J). Neuropathic pain symptoms did not reappear in PAC-treated rats one week (D36 or D37) after the end of 3α-DIOL corrective or prophylactic treatment (Figure 6). To verify that the beneficial action of 3α-DIOL against PAC-induced painful neuropathy is not due to a persistent sedative effect causing weakness and reflex slowing, we have used the openfield test to assess the motor capacity of control and PAC-treated rats after 3α-DIOL corrective or prophylactic treatment. As shown in Table 1, horizontal and vertical exploratory behaviors were similar in all tested groups indicating that 3α-DIOL corrective or prophylactic treatment did not reduce the rat motor activity.

**Table 1:** Effect of 3α-DIOL treatment (4 mg/kg/every 2 days) on the exploratory behaviors of control- and PAC-treated rats.

**3α-DIOL Treatment**	**Group**	**Total number of squares entered**	**Total number of rears on the wall**
**Corrective**	**VEH_crem_+VEH_hpc_**	327.25 ± 6.41	32 ± 1.22
	**VEH_crem_+3α-DIOL**	320.5 ± 5.55	30.25 ± 1.70
		ns1	ns1
	**PAC+VEH_hpc_**	317.5 ± 9.51	31.5 ± 1.66
		ns1	ns1
	**PAC+3α-DIOL**	313 ± 8.18	30.5 ± 0.87
		ns1,2	ns1,2
**Prophylactic**	**VEH_crem_+VEH_hpc_**	308 ±13.28	29.75 ± 1.11
	**VEH_crem_+3α-DIOL**	305.75 ± 7.87	30.75 ± 2.66
		ns1	ns1
	**PAC+VEH_hpc_**	295.75 ± 15.44	31.25 ± 1.65
		ns1	ns1
	**PAC+3α-DIOL**	293 ± 6.31	31 ± 1.58
		ns1,2	ns1,2

Total numbers of squares entered (± SEM) and of rears on the wall (± SEM) indicated in the table are representative of horizontal and vertical exploratory behaviors in the openfield test. For the corrective 3α-DIOL treatment schedule, PAC or VEH_crem_ was injected at D1, D3, D5 and D7; 3α-DIOL or VEH_hpc_ i.p. administrations started 8 days after the end of PAC treatment. For the prophylactic treatment schedule, 3α-DIOL or VEH_hpc_ i.p. was administered every 2 days from D1 while PAC or VEH_crem_ treatment, which started from D9, was administered at D9, D11, D13 and D15. Testing was performed at D28, i.e. 1 day after the 7^th^ 3α-DIOL injection in the corrective treatment schedule or 3 days after the 13^th^ 3α-DIOL injection in the prophylactic treatment schedule n=4 per group; ns1, not significant compared to VEH_crem_+VEH_hpc_; ns2, not significant compared to PAC+VEH_hpc_. VEH, vehicle; 3α-DIOL, 3α-androstanediol; PAC, paclitaxel.

### Effects of 3α-androstanediol against PAC-induced peripheral nerve functional deficit

**Figure 7:**
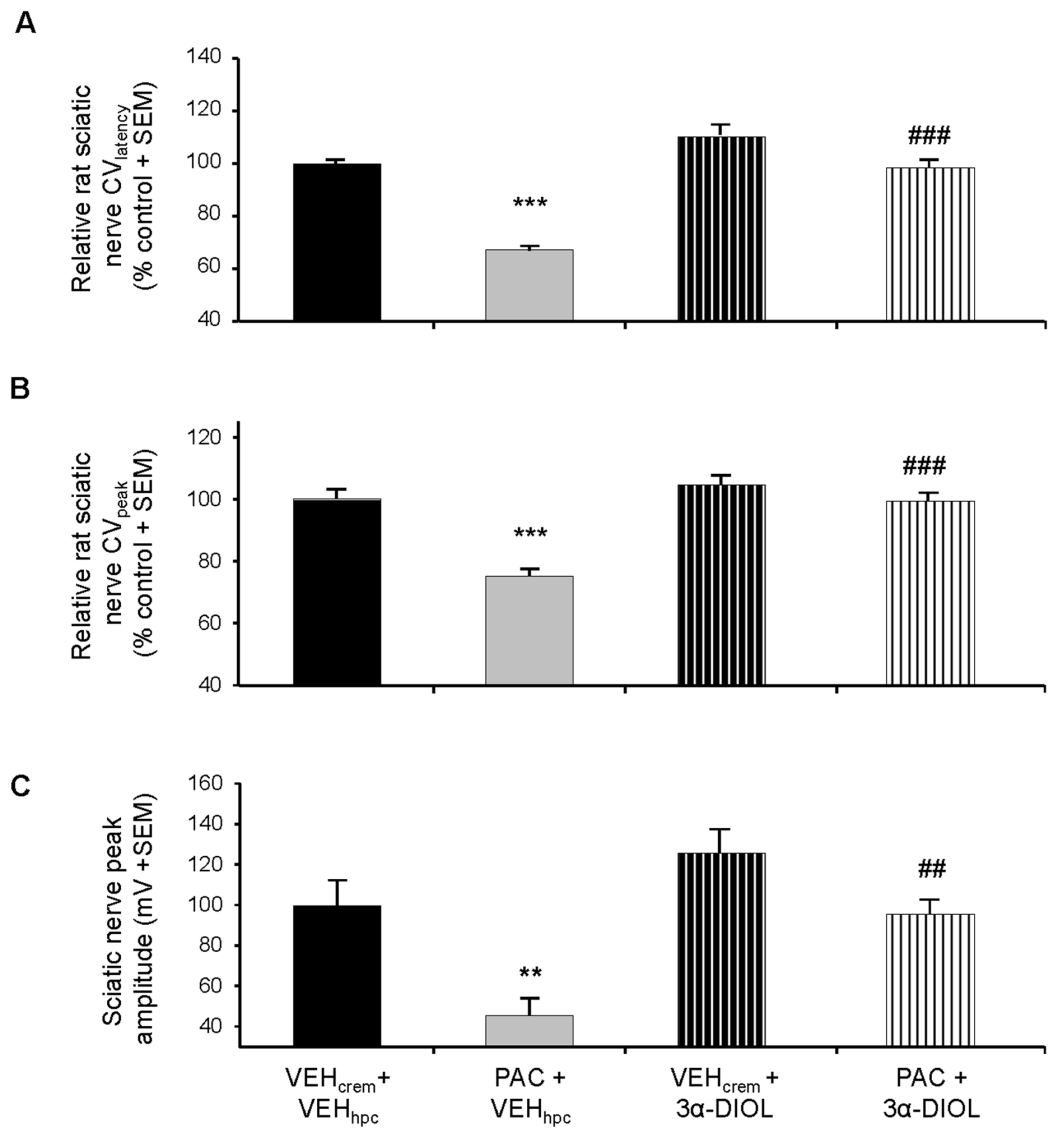
Curative effects of 3α-DIOL against PAC-induced reduction of sciatic nerve action potential parameters (CV_latency_: A; CV_peak_: B and peak amplitude: C). Histograms of normalized CV_latency_ and CV_peak_ show their reduction by PAC and recovery by 3α-DIOL treatment (**A**,**B**). Mean CV values were calculated as % of the mean CV obtained from vehicle-treated rats. (**C**) Recovery from PAC-induced NAP peak amplitude decrease by 3α-DIOL treatment. Each value is the mean (+SEM) of NAP peak amplitude obtained from 8 rat sciatic nerves per each group investigated. ** *p*<0.01, *** *p*<0.005. * vs (VEH_crem_ + VEH_hpc_), # vs (PAC + VEH_hpc_).

Treatment of naive rats with 3α-DIOL did not modify peripheral nerve activity even though an increase tendency of the mean values was observed in 3α-DIOL-treated rats compared to vehicle treated animals (Figure 7). Most importantly, 3α-DIOL reversed to normal values decreased CV_latency_, CV_peak_ and NAP peak amplitude induced by PAC treatment (Figure 7A-C). CV_latency_ (34.1 ± 1.1 m/s), CV_peak_ (13.0 ± 0.4 m/s) and NAP peak amplitude (1.5 ± 0.2 mV) mean values of (PAC+3α-DIOL)-treated rats were similar to the values of control animals (34.6 ± 0.6, 13.0 ± 0.4 m/s and 1.5 ± 0.1 mV, respectively).

### Effects of 3α-androstanediol on PAC-induced NF200 repression in peripheral nerve axons

**Figure 8:**
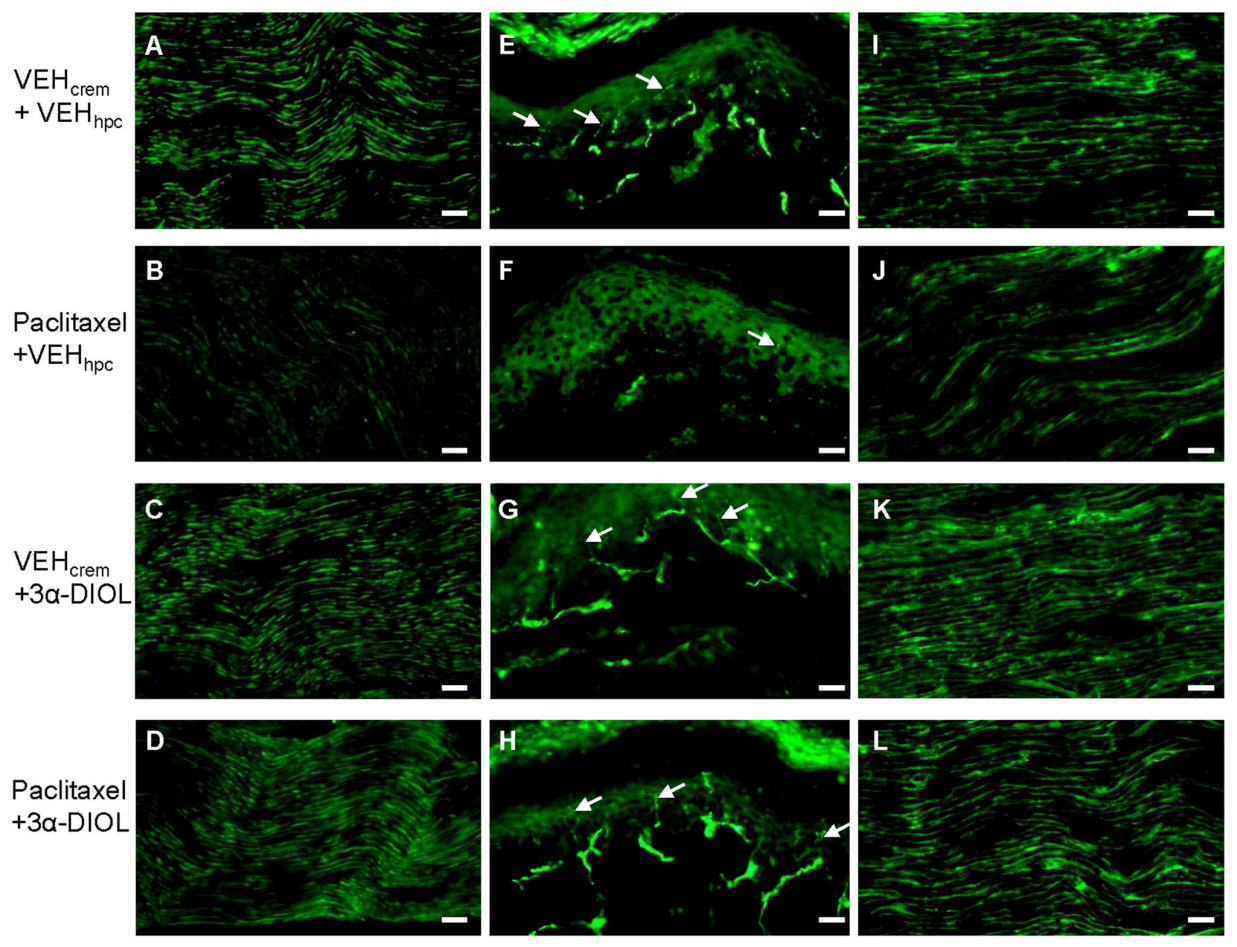
Photomicrographs of sagittal sections of sciatic nerves (A-D,I-L) or intraplantar skins (E-H) dissected from (VEH_crem_ + VEH_hpc_)(A,E,I)-, (PAC + VEH_hpc_)(B,F,J)-, (VEH_crem_ + 3α-DIOL)(C,G,K)- or (PAC + 3α-DIOL)(D,H,L)-treated rats. Nerve sections were labeled with the monoclonal NF200 antibody (**A**-**D**) or with the monoclonal anti-CNPase (**I**-**L**) revealed with Alexa-488-conjugated donkey anti-mouse. (**E**-**H**) Intraplantar skin sections were labeled with the polyclonal anti-PGP9.5 revealed with FITC-conjugated goat anti-rabbit. White arrows indicated intraepidermal nerve fibers. Scale bar, 50 µm.

**Figure 9:**
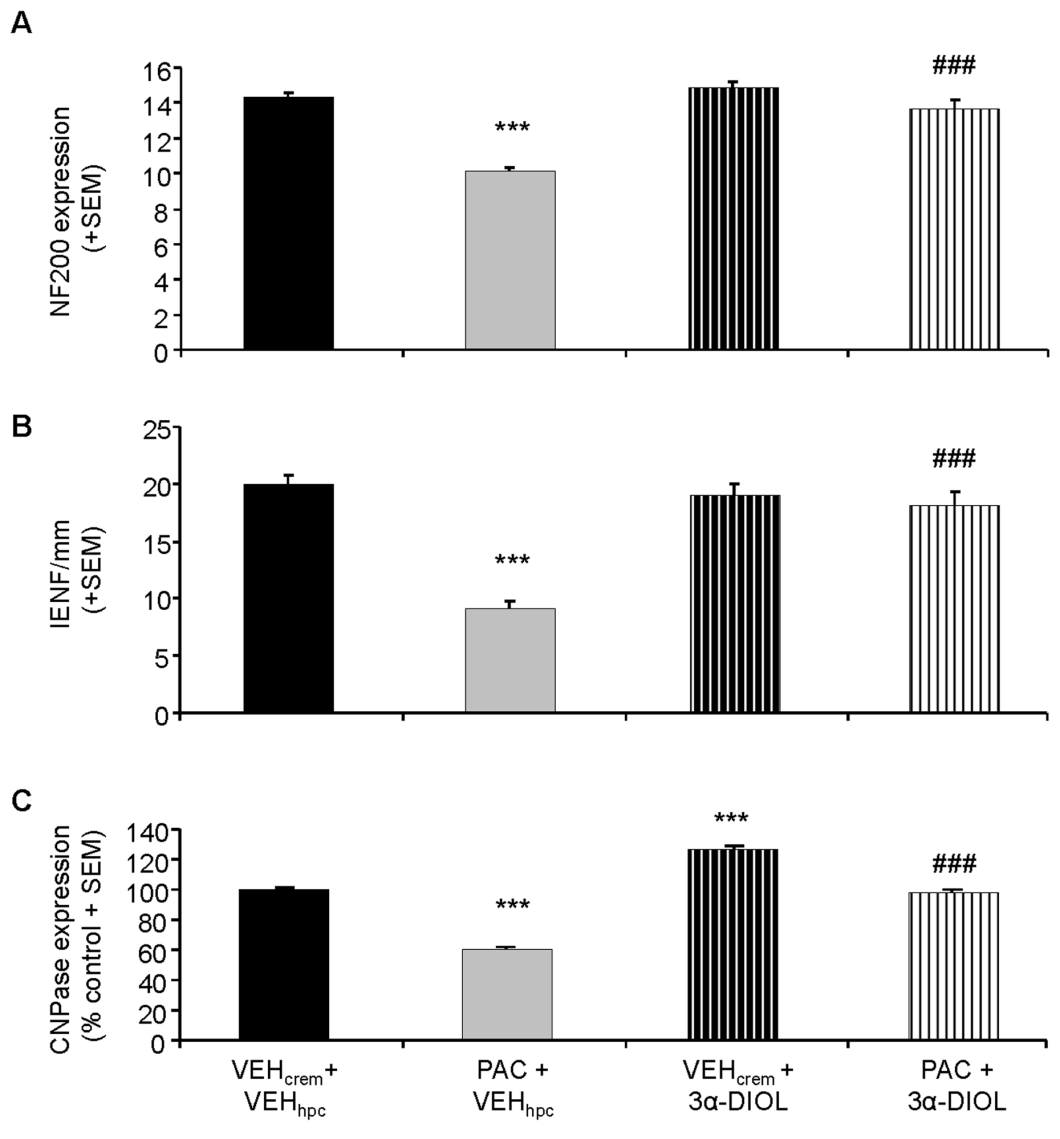
Neuroprotective effects of 3α-DIOL corrective treatment against PAC-induced alterations in sciatic nerves and intraplantar skin. (**A**-**C**) Chartbars show NF200 (**A**) or CNPase (**C**) expression level in sciatic nerve sections or IENF density in intraplantar skin sections (**B**) dissected from (VEH_crem_ + VEH_hpc_)-, (PAC + VEH_hpc_)-, (VEH_crem_ + 3α-DIOL)- and (PAC + 3α-DIOL)-treated rats. (**A**) Each value is expressed as mean (+ SEM) of actual counts for NF200-positive fibers detected in sciatic nerve sections. (**B**) Each value is expressed as mean (+ SEM) of IENF density (counts for PGP9.5-positive terminals) detected in intraplantar skin sections. (**C**) Each value is expressed as percent (+ SEM) of CNPase-positive cells bodies detected in sciatic nerve sections of control (VEH_crem_ + VEH_hpc_)-treated rats. n=8 per group. *** *p*<0.001 vs (VEH_crem_ + VEH_hpc_), ### *p*<0.001 vs (PAC + VEH_hpc_).

We observed that PAC treatment induced 30% decrease of NF200-immunoreactivity in rat sciatic nerves (Figure 8A,B and Figure 9A) (*p*<0.001). 3α-DIOL treatment administered during two weeks totally reversed to normal decreased NF200 expression level induced by PAC in rat sciatic nerve axons (*p*<0.001) (Figure 8A-D and Figure 9A).

### Effects of 3α-androstanediol on PAC-induced loss of intraepidermal nerve fibers

We detected 55% decrease of PGP9.5-immunoreactivity or IENF density in hind paw intra-plantar skins of PAC-treated rats compared to controls (*p*<0.001) (Figure 8E,F and Figure 9B). 3α-DIOL treatment during two weeks restored PGP9.5 or IENF normal density in PAC-treated animals (*p*<0.001) (Figure 8E-H and Figure 9B).

### Effect of 3α-androstanediol on CNPase expression in sciatic nerve

A strong increase of CNPase-immunoreactivity was visualized in sciatic nerves of 3α-DIOL- compared to vehicle- and PAC-treated rats (Figure 8I-L). In particular, counting of CNPase-positive cells revealed that 3α-DIOL induced a 26% increase (*p*<0.001) of CNPase expression in naive or healthy rat sciatic nerves (Figure 9C). Interestingly, the 40% decrease of CNPase-immunoreactivity evoked by PAC-treatment in rat sciatic nerves was totally abolished by 3α-DIOL treatment administered during two weeks (*p*<0.001) (Figure 8I-L and Figure 9C).

## Discussion

The present study revealed that a 7 day-treatment schedule with PAC (1 mg/kg, daily body weight) induced painful peripheral neuropathy and associated neurological symptoms in rats. In particular, we observed that PAC treatment alters functional, histological and neurochemical parameters of the rat peripheral nerves as previously described in humans and various other experimental models [1,3,12-18,58,59]. Indeed, after one week treatment with PAC, the treated rats developed physiological and functional abnormalities such as mechanical allodynia and hyperalgesia, cold allodynia, heat-thermal hyperalgesia, nerve conduction velocity decrease and NAP peak reduction. In addition, PAC also caused histopathological and neurochemical alterations including the repression of NF200 and CNPase in peripheral nerves and the decrease of IENF density in paw intraplantar skin. It is well known that NF200 is a constitutive protein of the cytoskeleton that pivotally controls neuronal cell morphology and axoplasmatic transport. Therefore, our results showing PAC-evoked NF200 repression in sciatic nerves suggest that PAC treatment may generate axonopathy in rats [1,3,60,61]. In agreement with this suggestion, neurophysiological investigation and peripheral nerve biopsy after docetaxel treatment in humans revealed an axonal neuropathy with a preferentially loss of large myelinated fibers [62]. Our studies also show that the natural neurosteroid 3α-DIOL is capable of preventing or correcting several major neuropathological symptoms evoked by PAC treatment. Indeed, our behavioral analyses demonstrated that prophylactic or corrective 3α-DIOL treatment prevented or suppressed PAC-induced neuropathic pain symptoms including cold allodynia, heat-thermal hyperalgesia and mechanical allodynia and hyperalgesia. Interestingly, our histopathological and neurochemical studies demonstrated that 3α-DIOL reversed to normal the repressed NF200 level observed in PAC-treated rat peripheral nerve axons, suggesting that 3α-DIOL-based therapy may be effective for the treatment of antineoplastic-evoked axonopathy and/or neuronopathy. Furthermore, our work revealed that 3α-DIOL prevented or counteracted PAC-induced PGP9.5-like immunoreactivity and IENF density decrease in intraplantar skin. Because epidermal innervation loss constitutes a neuropathological damage frequently occurring in antineoplastic-treated patients [1,52,63], 3α-DIOL appears as a promising neuroprotective compound against toxic drug-induced alterations of epidermal innervations.

It is well demonstrated that painful neurological symptoms such as allodynia and hyperalgesia evoked by anticancer drugs are generally associated with decreased peripheral nerve conduction velocity and ectopic discharges [4,13,17,60,64-66]. Consistently, our electrophysiological and behavioral investigations clearly evidenced a significant CV_latency_ reduction, NAP peak amplitude decrease and a marked allodynia and hyperalgesia in PAC-treated rats. Interestingly, 3α-DIOL was also capable of rescuing PAC-treated rats from these electrophysiological and functional alterations evoked by PAC treatment. Moreover, in NF knock-out mice, decreased NAP peak amplitude has been evidenced together with internodal conductance changes that are known to be responsible for nerve conduction block and CV reduction [67]. Low NF expression levels, which are major intrinsic determinants of axon caliber in large fibers, led to axonal diameter reduction and subsequently to CV slowing [67-70]. In perfect accordance with these data, our findings showed a drastic reduction of NF expression level in PAC-treated rat peripheral nerves that was correlated to a significant decrease of large fiber CV and NAP peak amplitude. Therefore, the neuroprotective effect of 3α-DIOL against PAC-induced painful neuropathological symptoms appears to be dependent on its ability to reverse to normal values decreased NF expression level, reduced CV_latency_ and NAP peak amplitude in PAC-treated rat peripheral nerves.

Moreover, our results also revealed that 3α-DIOL modulate differentially the expression of CNPase, NF200 and PGP9.5 genes. Indeed, in naive or vehicle-treated rats, 3α-DIOL increased CNPase expression level in peripheral nerves but did not modify the amounts of NF200 and PGP9.5. Interestingly, under neuropathological situations when NF200, PGP9.5 and CNPase levels were repressed by PAC treatment, 3α-DIOL was able to restore to normal the levels of all of these 3 key proteins controlling peripheral nerve activities and intraepidermal innervation sensory functions. Altogether, these results suggest that CNPase gene expression may be highly sensitive to 3α-DIOL-evoked intracellular signaling while the sensitivity of NF200 and PGP9.5 genes to 3α-DIOL may increase only under pathophysiological situations thanks to the activation of various transcription factors evoked by PAC treatment [71-73]. Also, 3α-DIOL is known to stimulate the central inhibitory system via the GABA_A_ receptor, suggesting that 3α-DIOL may exert an analgesic action which may contribute to suppress PAC-evoked painful symptoms [26-31,46]. Another important point is the fact that 3α-DIOL may be inter-converted into 5α-DHT by the enzyme 3α-hydroxysteroid oxido-reductase abundantly expressed in the nervous system [39,74,75]. Therefore, the treatment with exogenous 3α-DIOL may generate substantial endogenous concentrations of 3α-DIOL and 5α-DHT which can exert both neuroprotective and analgesic effects by activating GABA_A_ receptors and androgen nuclear receptors, respectively [27-29,31,32,34,46,76]. The genomic mechanisms activated by 3α-DIOL treatment (via its metabolite 5α-DHT) may explain 3α-DIOL ability to restore to normal values the decreased CNPase, NF200 and PGP9.5 expression levels evoked by PAC treatment in rat peripheral nerves. In support of this hypothesis, it has been shown that androgens, acting via their nuclear receptors, promote NF expression and neuritic process development in peripheral nerves [77]. Furthermore, it has also been demonstrated that, in rodents, androgens promote the expression of myelin proteins including CNPase level [78].

The beneficial effects exerted by 3α-DIOL against thermal and mechanical allodynic and hyperalgesic symptoms may result from the direct interactions of 3α-DIOL with GABA_A_ receptors inducing therefore and acute analgesia. However, several indications provided by the present study show that the beneficial action of 3α-DIOL against PAC-evoked painful neuropathy does not depend on 3α-DIOL-induced sedation or is not only the consequence of 3α-DIOL-evoked acute analgesia but results from the combination of 3α-DIOL neuroprotective ability, nerve tissue repairing capacity and long term analgesic property. Indeed, we observed that prophylactic 3α-DIOL treatment prevented neurochemical and functional alterations of peripheral nerves and IENF in PAC-treated rats and these animals did not exhibit painful neuropathological symptoms several days after the end of 3α-DIOL preventive administration. In addition, one week after withdrawal of 3α-DIOL corrective treatment, painful neurological symptoms did not reappear in PAC-treated rats. Finally, our openfield investigations revealed that the exploratory behavior and motor activity were similar in control and PAC-treated rats after 3α-DIOL preventive or corrective treatment, indicating that 3α-DIOL beneficial effects against PAC-evoked painful peripheral neuropathy and neurological symptoms did not result from its sedative action.

In conclusion, the present study, which shows that the neuroprotective neurosteroid 3α-DIOL effectively prevents and suppresses several painful and neuropathological symptoms evoked by PAC, also offers interesting opportunities for the development neurosteroid-based therapies against chemotherapy-induced peripheral neuropathy and painful neurological disorders.
